# Pathogenicity and virulence of *Mycoplasma genitalium*: Unraveling Ariadne’s Thread

**DOI:** 10.1080/21505594.2022.2095741

**Published:** 2022-07-05

**Authors:** Wu Yueyue, Xiu Feichen, Xi Yixuan, Liu Lu, Chen Yiwen, You Xiaoxing

**Affiliations:** Institute of Pathogenic Biology, Hengyang Medical School; Hunan Provincial Key Laboratory for Special Pathogens Prevention and Control; Hunan Province Cooperative Innovation Center for Molecular Target New Drug Study, University of South China, Hengyang, China

**Keywords:** *Mycoplasma genitalium*, cytadherence, membrane lipoprotein, immune escape

## Abstract

*Mycoplasma genitalium*, a pathogen from class Mollicutes, has been linked to sexually transmitted diseases and sparked widespread concern. To adapt to its environment, *M. genitalium* has evolved specific adhesins and motility mechanisms that allow it to adhere to and invade various eukaryotic cells, thereby causing severe damage to the cells. Even though traditional exotoxins have not been identified, secreted nucleases or membrane lipoproteins have been shown to cause cell death and inflammatory injury in *M. genitalium* infection. However, as both innate and adaptive immune responses are important for controlling infection, the immune responses that develop upon infection do not necessarily eliminate the organism completely. Antigenic variation, detoxifying enzymes, immunoglobulins, neutrophil extracellular trap-degrading enzymes, cell invasion, and biofilm formation are important factors that help the pathogen overcome the host defence and cause chronic infections in susceptible individuals. Furthermore, *M. genitalium* can increase the susceptibility to several sexually transmitted pathogens, which significantly complicates the persistence and chronicity of *M. genitalium* infection. This review aimed to discuss the virulence factors of *M. genitalium* to shed light on its complex pathogenicity and pathogenesis of the infection.

## INTRODUCTION

*Mycoplasma genitalium*, belonging to class Mollicutes, is the smallest prokaryotic bacterium capable of self-replication. It is a fastidious, slow-growing organism, with a genomic size of approximately 580 kb, comprising only 517 genes [1]. *M. genitalium* was initially identified in patients with non-gonococcal urethritis (NGU) in 1981, and it was later isolated from respiratory and synovial tissues as well [2–4]. *M. genitalium* is a human pathogen that is parasitic in the genitourinary tract and causes acute and chronic NGU in men, and cervicitis and pelvic inflammatory disease (PID) in women. Its prevalence rates are higher in patients attending sexual health clinics and in men with NGU, which is estimated to be 10–35% [5]. However, it is not a notifiable infection, there is insufficient data on its prevalence [6–9]. *M. genitalium* is also reported to for its capacity to bind to red blood cells[6] and co-infect with other pathogens, such as human immunodeficiency virus (HIV), *Chlamydia trachomatis*, *Neisseria gonorrhoeae*, and *Trichomonas vaginalis* [7]. Clinical data indicate that sexually transmitted infections by *M. genitalium* have become more serious worldwide, especially given their marked and rapid propensity for developing antimicrobial resistance [10]. Unfortunately, few antibiotics are available to treat *M. genitalium* infections, and there has been a marked rise in resistance to the antibiotics azithromycin and moxifloxacin, two antibiotics often used to treat *M. genitalium* infections [5,11–13]. Therefore, uncovering the pathogenic mechanism and virulence factors of *M. genitalium* will be greatly beneficial for therapeutic and vaccine development.

Similar to other pathogenic microorganisms, the adhesion of *M. genitalium* to host cells or extracellular matrix is a prerequisite to overcome numerous obstacles before effectively breaking through the host defence. Adherence is a complex process that is, to a large extent, facilitated by adhesins and auxiliary proteins as well as certain glycolytic enzymes that act as adhesins [14,15]. These adhesins are also considered to be necessary for internalization into host cells. Moreover, it is worth noting that *M. genitalium* also possesses a unique gliding motility that involves a series of adhesins and adhesion-related proteins and enhances the ability of the pathogen to colonize and spread infection [15].

**Figure 1.**
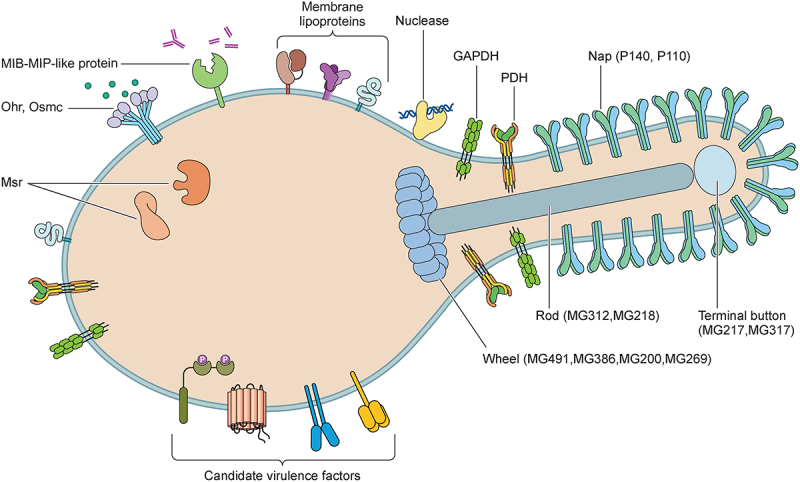
Diagrammatic representation of the pathogenic components of *Mycoplasma genitalium*. The pathogenesis and virulence of *M. genitalium* include invasiveness, toxic nuclease, and membrane lipoproteins. Numerous factors contribute to invasiveness, including proteins involved in adhesion, gliding motility, and antigenic variation, detoxifying enzymes involved in immune evasion (methionine sulphoxide reductase (Msr), organic hydroperoxide reductase (Ohr), osmotically inducible protein C (OsmC), and immunoglobulin proteases), and biofilms involved in antibiotic resistance and persistent infection. Additionally, glycosyltransferase, serine/threonine kinase, and serine/threonine phosphatase are considered candidate virulence factors of *M. genitalium.*

Owing to its inability to synthesize proteins, nucleotides, lipids, and sterols, *M. genitalium* obtains several nutrients from the host that the bacterium is unable to synthesize [8,14]; this parasitic mode of survival makes this organism heavily reliant on the host environment for essential nutrients to extend its survival time. Indeed, mycoplasma encodes nuclease to use nucleotide precursors for metabolic processes and propagation, which are considered to be necessary for initiating pathways leading to host cell death and subsequent pathological consequences [16]. Another important pathogenic mechanism of *M. genitalium* is the induction of inflammatory response in the urogenital tract primarily mediated by surfaced-exposed lipoproteins, resulting in inflammation and cellular damage [17]. Surprisingly, *M. genitalium* can proliferate and persist for a long time period after infecting a suitable host, even faced with a sophisticated immune system. To establish an infection, the cunning mycoplasma has a wide range of strategies to subvert the host immune responses, including antigenic variation, invasion of eukaryotic cells, cleavage of immunoglobulins (Igs), and biofilm formation, all of which are beneficial for the long-term survival of *M. genitalium* [17]. Chronic *M. genitalium* infection may raise the risk of co-infection with other pathogens, concurrently, the long-term interaction of *M. genitalium* with host cells may lead to a malignant transformation of the host cells [18]. This review aims to provide an overview of the virulence factors and pathogenic mechanisms that contribute to the pathogenicity of *M. genitalium* as well as the novel strategies identified in *M. genitalium*, including gliding motility, immunopathological injury, evasion of host immune responses, and potential carcinogenesis, all of which promote its colonization, propagation, and transmission *in vivo* in hosts (Figure 1).

## ADHERENCE TO HOST CELLS

Adherence is the first critical step in pathogenesis. Although it appears to be a regular process, as is well known, adherence is not a direct and unitary process [19]. *M. genitalium* has a flask-shaped appearance owing to the protrusion of the cellular membrane, termed as the attachment organelle or terminal organelle. The attachment organelle has a complex structure associated with the adherence potential of *M. genitalium* [20] and has been observed in several mycoplasma species. Currently, the most extensively studied attachment organelle and adhesin are those in *M. pneumoniae* and *M. genitalium*. The former primarily parasitizes the human respiratory tract and is associated with primary atypical pneumonia, whereas the latter grows extensively in the human genital tract and is associated with NGU [19]. The adhesion organelle of *M. genitalium* is maintained by a complex cytoskeleton composed of three major substructures: the terminal button, rod, and wheel complex (or bowl), present in the tip, centre, and back, respectively [20,21]. Ultrastructurally, the terminal organelle with an electron-dense core is composed of numerous cytoskeletal proteins, heat shock proteins, energy metabolic enzymes, and most cytadherence-related proteins [19,22,23]. The cytoskeleton is critical for the functions of mycoplasma, because it provides cells with a defined morphology [22]. Given that the terminal structure of *M. genitalium* is similar to that of *M. pneumoniae*, we speculate that cytadherence-related proteins may be involved not only in the clustering and anchoring of cytadhesins, but also in the maintenance and integrity of the terminal organelle [19,24].

Of note, the close interaction between the surface of epithelial cells and *M. genitalium* is typically related to adherence throughout the cell body, rather than only at the terminal organelle. The “Nap,” a transmembrane dimer of the surface adhesins P110 and P140, can be observed at the terminal organelle using electron microscopy [17]. Aparicio et al. used cryo-electron microscopy and tomography to show that Nap can exist in a “closed” or “open” conformation, which may lead to structural rearrangements and is crucial for cytadherence and release to cell receptors [6]. The unique terminal Nap-covered structure plays an essential role in *M. genitalium* adherence to various eukaryotic cells [6,24,25], as evidenced by the polarity of mycoplasma terminal organelle-mediated adhesion events and the nature of some adhesin proteins, such as P140 [24]. Additionally, the Nap adhesion complex participates in a unique type of gliding motility in *M. genitalium* via a conformative alternation-dependent mechanism [6,25]. Furthermore, Nap exhibits strong immunoreactivity as the primary target of antibody reactions; however, it is worth noting that P140 and P110 are susceptible to antigenic variation, which allows them to evade host immune recognition [6,26]. Notably, although *M. genitalium* has a relatively small genome, a group of hypothetical genes encoding proteins with novel properties have been characterized over time, and these are assumed to act as elements of the terminal organelle, which confirms the distinctiveness of the unique structural arrangement [15,27] (Table 1).

**Table 1.** A summary of *Mycoplasma genitalium* adhesion and motility-related proteins.

Proteins	Localization	Adherence	Motility	References
P140	Transmembrane	+	+	[19,24,28]
P110	Transmembrane	+	+	[19,28]
P32	Surface-exposed	+	Unclear	[29]
GAPDH, PDH	Surface-exposed	+	-	[14]
MG218	Rod	+	+	[23,30,31]
MG317	Terminal button	+	+	[21,23]
MG312	Rod	+	+	[15]
P69	Unclear	Unclear	Unclear	[29]
MG200	Wheel	Unclear	+	[28,32]
MG386	Wheel	Unclear	+	[28,32]
MG491	Wheel	Unclear	+	[20,28]
MG219	Unclear	Unclear	+	[33]
MG217	Terminal button	Unclear	+	[34]
MG269	Wheel	Unclear	+	[21]

### Adhesins involved in pathogenesis

P140 and P110, the two major *M. genitalium* adhesins, have been identified as virulence factors [15,27]. P140 exhibits high sequence homology and a wide range of immunological cross-reactivities with the P1 cytadhesin of *M. pneumoniae*. P140 and/or P110 mutants exhibit distorted morphologies, hypoplasia of terminal organelles, and loss of adhesion [27,30,35]. Although not as critical as P140 and P110, other adhesins, such as P32, are also of significant importance in the adherence and motility of *M. genitalium* [29]. Additionally, recent findings indicate that the metabolic enzyme glyceraldehyde-3-phosphate dehydrogenase (GAPDH) acts as an adhesin for *M. genitalium* [14]; this shows that the efficient adhesion of *M. genitalium* is far more complex than previously known.

#### P140

The adhesin P140 (also known as MgPa), which is encoded by *mgpB* (or *mg191*), is a surface-exposed trypsin-sensitive protein predominantly present in the terminal organelle and is required for the invasion of host cells by *M. genitalium* as well as for the human immune response [19,24]. *M. genitalium* is considered to adhere to host cells predominantly via P140. Mutation in P140 results in a cytadherence-negative phenotype, emphasizing the importance of P140 in *M. genitalium* pathogenesis [19]. P140 is a transmembrane adhesin that contains a large *N*-domain and a small C-domain, followed by a transmembrane helix required for attachment to host cells [25]. The *N*-terminal domain consists of a seven-bladed (β-sheet) propeller and a “crown” formed by the clustering of long polypeptide segments that emerge from the propeller [6,25]. Interestingly, the *N*-domain of P140 likely represents the signal peptide, as it corresponds to the predicted signal sequence, and the end of N-domain contains a few disordered residues. The C-domain of the extracellular region of P140 shows low sequence variability, and the conserved C-domain is separated into two distinct domains by a transmembrane region by model prediction: one extracellular and the other intracellular, which are referred to as “proximal” and “distal,” respectively. The distal fragment is abundant in conserved proline residues, which provides rigidity to the C-domain. The C-domain is considered to play an important role in Nap, acting as a stalk to anchor the adhesin to the mycoplasma membrane. Furthermore, a malleable region has been identified in the C- domain, which is sensitive to interaction with the N-domain, whereas the transmembrane helix connects the region free from the influence of N-domain interactions [36]. It was found that the N- and C-domains move relative to one another and act as important components in the conformational transitions of Nap during adhesion to host receptors [25,36,37]. Owing to the high homology between P140 of *M. genitalium* and P1 adhesin of *M. pneumoniae*, these cross-reactive epitopes show biological and immunological similarities [31,38]. Of note, P140 tends to undergo variation to evade host immune recognition, as discussed below.

#### P110

P110 (also known as MgPc or P114), which is encoded by *mg192* or *mgpC*, is an immunoreactive surface-exposed adhesin primarily present in the terminal organelle. Although P110 and P140 show a high degree of homology at their distal cytoplasmic C-termini, they do not show cross-reactivity. In *M. genitalium*, P110 is a cytadherence-associated protein required for the stability of the primary adhesion P140 [19]. The extracellular region of P110 also contains a large *N*-domain and a small C-domain. The *N*-domain has a topology consistent with a seven-bladed β-propeller, whereas the C-domain contains a previously unidentified compact fold with a β-barrel of five antiparallel strands, a β-hairpin, and four small α-helices. P110 shares many features of domain organization and the topology of the secondary structural elements with P140 [6]; the overall structure and domain organization of the P110 extracellular region forms a shape similar to the body and the stalk of the uppercase letter “P” [19,27]. Within the *N*-domain of P110, the connections between some blades or β-strands create a “crown” structure that contains a binding site for sialic acid oligosaccharides [39]. Crystal structure shows that the binding site for the sialylated oligosaccharides is localized at the interface between P140 and P110 in the N-terminal domain of P140–P110 complex. In this structure, P110 residues Gln460–Asp461 and Arg600 play an important role in the interaction with P140 [6,27]. It was concluded that P110 has a highly variable sequence in both *in vitro* cultured organisms and *in vivo* specimens, which allows *M. genitalium* to undergo antigenic variation or host immune evasion [40,41]. In addition to supporting P140, P110 has a homolog and exhibits cross-immunity with the *M. pneumoniae* P90/P40 pair [27,42].

P110 and P140 adhesins are known to be reciprocally stabilized and shown to exhibit a mutual post-translational stabilization effect; for example, the mutation of either P110 or P140 decreased the expression of the other adhesin [19]. Additionally, P140 and P110 can modulate the expression of other proteins. For instance, when P140 or P110 are absent or depleted, the level of MG386 is decreased, whereas the expression of MG305 (DnaK) is increased [19]. The upregulation of DnaK in the absence of P140/P110 could be considered as the trigger for a stress signalling response in *M. genitalium*, closely resembling that observed after a heat shock. Of course, we cannot rule out the possibility that increased DnaK expression results from cytoskeletal rearrangements under the absence of the attachment organelle [19].

#### P32

P32, encoded by *mg318*, is a membrane-associated surface-exposed adhesin present at the distal end of the attachment organelle. It is analogous to the P30 adhesin of *M. pneumoniae*. Both P32 and P30 possess novel proline-rich repeats at their C-termini, although there are differences in repeats that are less regular in P32 [29,43–45]. P32 is presumed to contribute, at least partially, to the stability of the major adhesins P140 and P110, as the transfection of an additional copy of the *mg318* gene into both MG218 and MG491 null mutant strains significantly restored the P140 and P110 levels [28,43]. Since *M. pneumoniae* P30 adhesin-deficient protein shows a noncytadhering, avirulent phenotype and abnormal tip structures [46], we may assume that *M. genitalium* P32 performs a similar function.

#### GAPDH and pyruvate dehydrogenase (PDH)

The finding that glycolytic enzymes act as mycoplasma adhesins can be traced back to the early 2000s, when alternative adherence pathways, apart from the tip-mediated cytadherence of *M. genitalium*, were investigated [14]. Three mucin-binding proteins from *M. genitalium* were identified through continuous efforts; one of these was identified as GAPDH. *M. genitalium* was shown to be capable of adhering to mucin-coated microtiter wells in an *in vitro* assay, and GAPDH was confirmed to serve as an adhesin. As an important enzyme of the glycolysis pathway, the primary functional domain of GAPDH is localized in the cytosol. However, further analysis revealed that 10% of the total GAPDH was localized to the membrane, although the concentration of surface-exposed GAPDH varied among *M. genitalium* subpopulations [14]. The differences in GAPDH profile relocation between individual mycoplasmas may govern the ability of *M. genitalium* subpopulations to adhere to or invade target cells.

The other two mucin-binding proteins identified were PDH subunits A and B (PDH-A and PDH-B); their presence could explain why GAPDH epitope blockade by antibodies only reduces the binding of *M. genitalium* to mucin by 70% [14]. These findings established the fact that the relocation of glycolytic enzymes to the *M. genitalium* membrane surface imparts completely independent and unforeseeable properties, such as mucin-binding activity. Given that the *M. genitalium* genome is considerably small, its ability to exploit its restricted genome may be explained in part by the various functions of its proteins, mediated via distinct compartmentalization and localization changes. The ability of *M. genitalium* to bind to mucin via cytoplasmic proteins (e.g. GAPDH, PDH-A, and PDH-B) may be a vital mechanism, using which mycoplasmas circumvent genetic restrictions, which facilitates the colonization and invasion of host tissues. The multifaceted roles of GAPDH and PDH enhance their biological and biochemical potential, while also partially influencing *M. genitalium* pathogenesis.

### Accessory proteins

Major adhesin proteins interact with accessory proteins to form a complete attachment organelle structure. Compared to adhesins, which interact directly with the host cell, accessory proteins may not play a direct role in adhesion [47]. However, mutations in accessory proteins can lead to the loss or reduction of adhesion ability, and restoration of their expression can help regain the cytadherence-positive phenotype. *M. pneumoniae* and *M. genitalium* accessory proteins are the most extensively studied among the accessory proteins of human pathogenic mycoplasma. Numerous studies have suggested that multiple putative accessory proteins, such as MG218, MG317, MG312, and p69, play essential roles in promoting the translocation and positioning of adhesins, such as P110 and P140, on the surface to form an “attachment organelle” and eventually complete the cytadherence process [45,47]. Owing to the close association between cytadherence and motility, numerous cytoskeletal proteins, such as MG218 and MG317, are implicated in both adherence and motility.

#### MG218

The cytadherence-related protein MG218 is encoded by *mg218*, the largest open reading frame in the *M. genitalium* genome. Mature MG218 is predicted to be a 216 kDa protein largely similar to the *M. pneumoniae* accessory protein HMW2. MG218 is a basic protein with several leucine zippers and a coiled-coil structure, which enables it to interact with various other proteins, thereby affecting the function of certain adhesion molecules. For example, the MG218 mutant shows post-translational defects, which may lead to the inability of the major adhesins P140 and P110 to maintain structural integrity. Additionally, evidence suggests that the C-terminal region and the 330 amino acids of the *N*-terminus of MG218 are required for P140/P110 stability [30,31], but do not exert direct transcriptional or translational impact on the P140 operon, implying that additional factors are required for the efficient expression of P140 and P110 in addition to the auxiliary expression of MG218 [30].

#### MG317

MG317 shares DNA sequence homology with the cytadherence-related protein HMW3 of *M. pneumoniae* [23]. MG317 mutants show an abnormal electron-dense core and reduced cytadherence and gliding motility, indicating that MG317 is necessary for the formation of the terminal button and helps anchor the electron-dense core to the cell membrane. Additionally, studies have indicated that *mg317* and *mg218* mutants show lower P140 and P110 expression, a negative-hemadsorption (HA-) phenotype, fewer motile cells, and a lower gliding speed, in addition to abnormalities in the terminal organelle. Interestingly, the loss of *mg218* and *mg317* expression has also been implicated in colony development [23]. Therefore, MG317 seems to play a critical role in the terminal organelle architecture and is an important component in the maintenance of cytadherence and gliding motility in *M. genitalium*, almost similar to MG218.

#### MG312

MG312 is a homolog of *M. pneumoniae* HMW1. It is composed of highly conserved C- and *N*-terminal domains joined by a less conserved central domain comprising repeating acidic and rich proline motifs. *mg312* deletion leads to a reduced HA phenotype, abnormal cell morphology, and loss of attachment organelles, along with impairment of adherence and gliding ability. Further investigation revealed that the C-terminal domain of MG312 is necessary for *M. genitalium* terminal organelle formation. The loss of MG312 expression may affect the stability of the primary components of the terminal organelle, P140 and P110, as well as of MG386, MG218, MG217, and MG317, indicating that MG312 is involved in the maintenance of the integrity of the terminal organelle in *M. genitalium* [15].

#### P69

As with MG218, *M. genitalium* P69 is highly homologous to *M. pneumoniae* HMW3. Notably, P69 was the first adherence-accessory protein homolog identified in *M. genitalium*. In a hamster model, *M. pneumoniae* mutants lacking HMW3 expression were unable to achieve cytadherence and displayed a loss of virulence [29,48]. Therefore, the precise function of P69 requires further investigation.

### Etiological significance of adhesion

The pathogenesis of *M. genitalium* relies heavily on its adhesion. *In vitro*, *M. genitalium* adheres to multiple eukaryotic cells, such as Vero monkey kidney cells and Hep-2 cells, based on its terminal structure [8]. Moreover, it is capable of adhering to both cilia and fallopian tube epithelial cells in cultured organs [8,49]. A more precise explanation of the adhesion mechanism will help determine the myriad processes underlying *M. genitalium* pathogenesis and aid clinical prevention.

A host cell can be damaged further once *M. genitalium* is attached to its surface. For example, *M. genitalium* is capable of binding to human spermatozoa and can thus be transported by motile spermatozoa. Male infertility may be caused by injury to *M. genitalium*-bound sperm, such as that resulting from agglutination and immobility. The motile sperm appears to carry *M. genitalium*, which then colonizes and destroys the ciliated epithelia in the uterus and fallopian tubes, leading to genital diseases and infertility in females [49,50].

Another critical outcome of *M. genitalium* adhesion is internalization into the host cell. Direct interaction between *M. genitalium* and the host cell membrane is considered to be essential, and the loss of adhesion activity reduces the natural invasiveness of *M. genitalium* [51]. Apart from the pathogenic attachment of *M. genitalium*, which causes host cell damage, a severe consequence of invasion is the ability of *M. genitalium* to survive for extended periods in invading host cells, which is an important cause of chronic persistent infection and also poses significant challenges in treatment. The mechanism underlying sustained survival will be discussed below [8,51].

## GLIDING MOTILITY

Motility is frequently recognized as a crucial pathogenic factor for several bacteria, as it facilitates the transmission of infection and increases the ability of pathogens to colonize target cells [34]. *M. genitalium* lacks specialized motility structures, such as flagella or pili, and no homologs to other bacterial locomotive organs have been identified in this organism. *M. genitalium* motility is believed to be mediated by a novel and unknown mechanism that allows its penetration into the mucous layer, which exerts a protective action on mucosal epithelial cells [32,43,52]. Indeed, *M. genitalium*, similar to other gliding mycoplasmas, is known to move across solid surfaces in a unique manner, which is referred to as gliding motility. Gliding motility is a method of moving across surfaces without the assistance of obvious motility organelles. Gliding motility has been observed in several eubacteria, including *M. pneumoniae* and *M. gallisepticum*. Evidently, gliding motility is closely linked to the terminal organelle. Evidence indicates that the terminal organelle not only supplies power, but also acts as a guiding system when the cell pulls forward [33,53]. Although the exact mechanism by which terminal organelles mediate motility needs further investigation, gliding motility is known to represent a unique type of motility characterized by the catch, binding, and release activity of the P140/P110 complex [17,28]. Understandably, mutations in P110 or P140 may cause the loss of cytadhesion as well as a deficiency in motility [15,32]. Therefore, gliding motility is important for the colonization and dissemination of *M. genitalium* pathogenesis and acts as a pivotal virulence factor in this organism.

The attachment organelle of *M. genitalium* has a length of only 170 nm and curves to approximately 20°, with a more prominent terminal knob. In comparison, *M. pneumoniae* has a straight attachment organelle, but of a greater length (290 nm), owing to the presence of different core substructures specific to the organism; this may partly explain the fact that the speed of gliding motility varies from species to species [32,43]. Furthermore, the function of terminal organelle core components is clearly related to the appropriate regulation and transmission of movement to the cell body than to movement generation [51]. Strains deficient in important cytoskeletal proteins (e.g. MG491, MG218 and MG318) remain capable of generating motile filamentous cells, demonstrating that certain cytoskeletal proteins are necessary but insufficient for movement generation [28].

### Proteins involved in motility

It has become increasingly clear that motility is a complex coordination process involving a wide range of adhesion-related proteins. At present, most studies have focused on the gliding motility-specific processes of *M. pneumoniae*; however, the mechanism underlying the process in *M. genitalium* is yet to be completely understood. Nonetheless, a subset of proteins involved in *M. genitalium* motility has been characterized. Among the proteins, certain cytoskeletal proteins (MG217, MG312, MG218, and MG317) appear to facilitate gliding motility by improving movement transmission to the host cell, stabilizing adhesin complexes and regulating the direction of cell movement. Although their function is unknown, they may contribute significantly to the motility of *M. genitalium* and, therefore, act as a powerful boost for host invasion [28,33].

#### MG200 and MG386

MG200 and MG386 play an important role in gliding motility; some homologs of MG200 and MG386 have been identified in other gliding mycoplasmas, such as *M. pneumoniae* and *M. gallisepticum* [32]. MG200 is considered a multi-domain protein, and the C-terminal region of MG200 lacks obvious sequence homology with other proteins, whereas the *N*-terminal segment contains a J-like domain that is responsible for regulating the activity of the molecular chaperones Hsp70 and DnaK, usually present in the DnaJ family [28,33]. MG386 and MG200 mutants showed varying degrees of gliding deficiencies, and the restoration of MG200 or MG386 expression in these mutants was found to restore the gliding ability of *M. genitalium*, which is direct evidence that MG200 and MG386 play important roles in gliding. Both MG200 and MG386 exhibit structural similarities. For example, both proteins have aromatic and glycine residue-rich boxes as well as an acidic and proline-rich domain, which are considered necessary for gliding motility [32]. Although MG386 and MG200 participate in gliding motility rather than directly affecting cell adhesion, the underlying mechanism and physiological significance warrant further investigation. MG200 appears to be implicated in adhesin folding and translocation to the cell membrane [28,32]. Alternatively, adhesin expression following mycoplasma infection may contribute to the stability of proteins involved in gliding motility. For example, *mg191* and *mg192* mutants showed decreased MG386 levels, indicating that P110 and P140 adhesins are required for MG386 stability [19,28]. This further demonstrates that adhesin and gliding-related proteins are not expressed separately and are functionally connected in numerous ways.

#### MG491

MG491 is of significant importance in the functioning, organization, and stabilization of terminal organelles. Sequence alignment of MG491 revealed 53% overall homology with *M. pneumoniae* P41. MG491 contains a 60-residue *N*-terminal region, a 150-residue central three-helix bundle, and a fairly flexible C-terminal region. The C-terminal region of MG491 contains a 25-residue-long peptide that plays a special role in its interaction with MG200 [20]. The MG200-MG491 interaction exerts a specific effect on motility and cell morphology, which determines the infective capability of *M. genitalium*. The loss of MG491 expression causes abnormalities in both motility and cell morphology, which indicates the importance of the MG200-MG491 interaction in maintaining the integrity of the terminal organelle. Additionally, *mg491* can undergo a point mutation that may significantly decrease motility [20]. Furthermore, the significance of spreading deficiencies associated with the decreased infectivity indicates that MG491 is a virulence factor for *M. genitalium* [20,54].

#### MG219

MG219 is a homolog of *M. pneumoniae* P24 and is considered a new component of the motility machinery of *M. genitalium*, but its specific function remains unknown. Although MG219 is expressed at low levels in *M. genitalium*, mutation in or the downregulation of MG219 resulted in a phenotype with gliding deficiency. The underlying mechanism is currently unknown, and the presence of MG219 depends on regions other than the EAGR box of MG200, which may interact with MG219 [33] and eventually contributes to *M. genitalium* motility.

#### MG217

MG217 is an intracellular gliding-associated protein that contains a proline-rich domain and a leucine zipper motif. These domains facilitate protein-protein interactions and contribute to the gliding motion of *M. genitalium*. MG217 may be involved in the curvature of the terminal structure of *M. genitalium* cells at a functional level, as the absence of MG217 was shown to alter the morphology of the terminal organelle. MG217 is positioned at the distal end of the attachment organelle, situated between the plasma membrane and the terminal button, as observed by immunogold labelling [34]. Its positioning facilitates its contribution to the movement of cells in a specific direction. MG217 has also been implicated in the assembly of the gliding apparatus and in directing cells to glide in narrow circles. The importance of MG217 is evident from the fact that the terminal organelle-guided mycoplasma cell steer is associated with the rearrangement of the terminal organelle disposition. Indeed, it has been demonstrated that in the absence of MG217, cells retain the ability to glide, but their gliding path changes from a narrow circular track to an erratic or wide circular path. The fact that the sliding behaviour of MG217 mutants is accompanied by a change in the terminal organelle curvature confirmed the critical role of MG217 in motility [34].

#### MG312

MG312 is an intracellular cytoskeletal protein that contributes to the gliding motility of *M. genitalium* by preserving the integrity of the terminal organelle. As with MG200, the *N*-terminus of MG312 contains an EAGR box that is apparently involved in interactions among gliding motility-related proteins, such as MG218, MG317, and MG312. The deletion of the EAGR boxes from MG312 or MG200 caused a motility disadvantage in mycoplasma cells, which indicated the significance and specific role of EAGR boxes in motility [15,33]. However, the exact significance of this motif in gliding motility remains unknown. Additionally, studies have shown that the Walker A box in the *N*-terminal region of MG312 is a conserved ATP-binding site, which implies that MG312 may play a role in the gliding mechanism in addition to its scaffolding function [15]. Interestingly, the removal of the *N*-terminal region of MG312 reduced, but did not completely abolish, the gliding motility of *M. genitalium*, supporting the hypothesis that this region may contribute to the conformational changes that drive cell gliding or perform a regulatory function in certain activities necessary for gliding [15].

#### MG218 and MG317

The cytadherence-related proteins MG218 and MG317 also contribute to the gliding motility of *M. genitalium*, as both participate in the formation of the terminal organelle, which helps maintain the gliding motility. Several mutants of *mg218* and *mg317* showed decreased motile cell numbers and gliding speed, which emphasized the roles of the proteins in *M. genitalium* motility [23].

## CELLULAR DAMAGE & INJURY

### Nuclease

Community-acquired respiratory distress syndrome (CARDS), caused by *M. pneumoniae*, defies the conventional belief that mycoplasma does not produce secretory toxins; however, no similar genes have been identified in *M. genitalium*, and nuclease appears to be one of the few toxic enzymes produced by this organism [16,55]. *M. genitalium*, similar to other mycoplasmas, is capable of degrading host nucleic acids as a source of nucleotide precursors to support its development and pathogenicity. MG186, the only nuclease produced by *M. genitalium*, was discovered by Li et al. in 2010; based on its subcellular localization, it is a lipoprotein expressed on the surface of tip organelles and other regions [16]. It is a Ca^2+^-dependent membrane-associated nuclease. MG186 is composed of 250 amino acids and contains conserved amino acid motifs similar to those present in thermostable nucleases. These conserved amino acid motifs are necessary for Ca^2+^ binding and active-site conformation [8,16]. Amino acid homology analysis revealed that MG186 shares an amino acid sequence (residue 1 to 245) with Mpn133, the well-known *M. pneumoniae* nuclease, with a homogeneity of up to 39%. Interestingly, Ca^2+^ is required for the activity of recombinant MG186 (rMG186). Meanwhile, Mn^2+^, Zn^2+^, and chelating agents such as EGTA and EDTA play an inhibitory role in the nuclease activity of rMG186, whereas Mg^2+^ does not appear to influence it. Further analysis indicated that its degradation efficacy for plasmid DNA or double-stranded DNA is lower than that for single-stranded DNA or RNA, implying that rMG186 prefers to degrade single-stranded nucleic acids [16]. *In vitro* experiments also revealed that rMG186 treatment causes DNA degradation and induces apoptotic morphological changes, such as nuclear shrinkage, chromatin condensation, and ghost nuclei formation, in human endometrial cells. These nuclear events are considered to be caused by rMG186 internalization, as indicated by findings from subcellular localization and Lamin integration experiments. Additionally, *M. genitalium*, which shows intracellular invasion, preferentially localizes to the perinuclear region, and the surface-exposed localization of MG186 supports this observation [16]. Of note, infection, hormonal changes, and other environmental stressors in the host appear to affect the concentration of Ca^2+^, which may affect the nuclease activity of *M. genitalium* [16,56]. MG186 is considered to be critical for the biosynthetic functions and replication of *M. genitalium*. Pathogenicity not only leads to competition with the host for the nucleic acid pool, but also to the degradation of neutrophil extracellular traps (NETs) that facilitate immune evasion [8,16,57]. Therefore, MG186 appears to be a critical pathogenic factor in the colonization of *M. genitalium* cells, causing direct or indirect damage to the host cell, and possibly playing a role in the persistence of the infection [16].

### Membrane lipoproteins and persistent immune activation

M.*genitalium*-induced mucosal immunological reactions play a critical role in eradicating invading bacteria. It is well established that the immune response or inflammation frequently exerts dual effects and may cause immunopathological injury. As a result, the damage caused by *M. genitalium* is largely immune-mediated and results from the secondary cellular overactivation of the immune response to primary infection [8,58].

Epithelial cells are essential for mucosal immune reactions following *M. genitalium* infection. Urogenitally inoculated animals showed increased polymorphonuclear leukocyte exudation and a strong antibody response to *M. genitalium* infection. Both human vaginal and cervical epithelial cells cultured *in vitro* were found to be susceptible to *M. genitalium*, which led to the rapid invasion of epithelial cells by *M. genitalium* and induced a cascade of inflammatory responses [8,59]. Although it has been recognized that *M. pneumoniae* infection may cause certain autoimmune diseases, and a cross-immune reaction is exhibited by *M. genitalium* and *M. pneumoniae* owing to the presence of shared antigens, the possibility that *M. genitalium* may cause autoimmune diseases cannot be ruled out, even though sufficient evidence is lacking [8].

Infection with *M. genitalium* frequently results in persistent chronic inflammatory syndromes. Persistence in lower tract tissues is one of the risk factors for PID and tubal-associated infertility, both of which occur in the upper reproductive tract. *M. genitalium* infection has been shown to be associated with chronic leukocyte infiltration and proinflammatory cytokine secretion. Neutrophils appear to be the most prevalent leukocytes in the cervix during chronic infection and are likely to contribute to immunopathogenic consequences and mucosal health [60]. For instance, persistent colonization of the urogenital mucosae may cause long-term inflammation and epithelial damage, thereby increasing the risk of transmission of other sexually transmitted infections. Although chronic infection induces persistent cytokine synthesis, there is no evidence of host cell cytotoxicity in women with low *M. genitalium* loads [61], implying that when persistent infection occurs, direct damage exerts a negligible effect on host cell integrity.

#### Membrane lipoproteins

In the absence of specific toxins or secretory virulence factors, *M. genitalium* evokes inflammatory responses primarily through multiple surface-exposed lipoproteins [17]. Given that mycoplasmas lack a rigid cell wall, the cell membrane has been recognized as the only structure using which mycoplasmas interact with host cells [62]. Studies published as early as 2008 demonstrated that the macrophage-stimulating activity of the Triton X-114 fraction of *M. genitalium* is linked to NF-κB activation via Toll-like receptor (TLR) 2, indicating that the lipid components of the *M. genitalium* membrane extracts mediate the inflammatory response [63]. Traditionally, membrane proteins can be classified as integral, peripheral, and lipid-anchored, with most mycoplasma membrane lipoproteins being lipid-anchored. Membrane lipoproteins are crucial for the pathogenesis of mycoplasmas. On one hand, certain adhesins are lipoproteins that are important for HA and surface adherence. On the other hand, the abundance of lipoproteins on the surface creates a protective layer around mycoplasma, thereby allowing it to evade the host antibody attack. Furthermore, lipoproteins act as strong pyrogens, which activate the host immune system and trigger an inflammatory response that causes tissue damage. Therefore, mycoplasma lipoproteins have been recognized as critical virulence determinants for *M. genitalium* [64].

Similar to bacterial lipoproteins, *M. genitalium* lipoproteins contain an S-diacylglyceryl-cysteine residue at their *N*-termini. In most mycoplasmas, the NH_2_ in cysteine exists in its free form, which causes the diacylation of lipoproteins [65]. This structure determines the specific TLR involved in lipoprotein recognition. Because of the absence of a specific gene encoding the *N*-acyltransferase (*Lnt*) enzyme, most mycoplasma lipoproteins were previously classified as diacylated lipoproteins [66]. Although no *Lnt* or *Lnt* homolog has been identified in *M. genitalium*, the discovery of the triacylated lipoprotein MG040 showed that certain proteins function as *Lnt* in *M. genitalium*, causing an additional acyl group transfer to the amino group of the diacylated cysteine residue. Clearly, the synthesis of mature MG040 warrants further investigation [64,65]. Lipoproteins present in *M. genitalium* are most likely involved in immunomodulation, adhesion, biofilm formation, and invasiveness in host cells as well as in nucleotide metabolism and ABC transporter function [67]. Most significantly, they can interact with TLRs expressed on innate immune cells to induce an inflammatory response.

#### Interaction of lipoproteins and TLRs

For a long time, the interaction between lipoproteins and TLRs has garnered significant attention. Similar to those in other mycoplasmas, the diacylated lipoproteins of *M. genitalium* are primarily recognized by the TLR2/6 heterodimers of the innate immune system. Notably, the TLR1/2 heterodimer was demonstrated to be involved in the recognition of the *M. genitalium* lipoprotein MG149, which indicates the existence of triacylated lipoproteins [63]. Owing to the absence of direct evidence from mass spectrometry or other structural analyses methods, such as nuclear magnetic resonance, and the absence of *Lnt* or orthologs in *M. genitalium* genomes (based on findings reported to date) [63,66], caution should be exercised when drawing such conclusions. However, triacylated lipoproteins (MG040) have been characterized in *M. genitalium* [65]. It remains unknown whether this lipoprotein is recognized by the TLR1/2 heterodimer and, thus, promotes inflammation.

The most prominent pathogenic effect of membrane lipoproteins is the proinflammatory response, which contributes significantly to the formation of lesions in the genital tract [68,69]. Although *M. genitalium* encodes various membrane lipoproteins, their immunostimulatory actions are distinct [63]. Thus, characterization of the active components of membrane lipoproteins is critical for understanding the proinflammatory mechanism adopted by *M. genitalium*. In early 2009, McGowin et al. showed that the C-terminus of recombinant MG309 (rMG309c) can activate NF-κB via TLR2 and TLR6 to induce the secretion of cytokines, including IL-6 and IL-8, from genital epithelial cells, thereby triggering an inflammatory response [69]. Later, Shimizu et al. demonstrated that when HEK293T cells were incubated with the Triton X-114 phase of *M. genitalium* membrane extracts, fractions matched to *M. genitalium* MG149 showed the highest NF-κB-inducing activity. Further investigation showed that MG149 can trigger TLR1 and TLR2 to activate NF-κB [63]. Based on evidence from subsequent *in vitro* and *in vivo* studies, it is now widely accepted that the components of the downstream signalling pathway following TLR2 activation (in the form of a TLR2/6 or TLR1/2 heterodimer) are comparable to those of TLR4 (such as mitogen-activated protein kinases, NF-κB, and AP-1) after the recruitment of the adaptor MyD88 and Mal, which ultimately causes multiple types of cells, such as cultured human vaginal and cervical epithelial cells, to generate proinflammatory cytokines, including but not limited to IL-6, IL-7 and IL-8 [8,45,60,63]. These cytokines have different biological activities and form complex synergistic or antagonistic networks *in vivo*, which determine the outcome of inflammation. It is known that NF-κB activation is integral for the innate immune response, and understandably, the persistent activation of vaginal epithelial cells by *M. genitalium* membrane lipoproteins could represent an important mechanism underlying genital inflammation [69].

## EVASION OF HOST IMMUNE SYSTEM & SUSTAINED SURVIVAL

Immune evasion is considered necessary for mycoplasma survival and persistent infection. *M. genitalium* has evolved various strategies for evading host immune responses, such as: (1) mutation of the major adhesins on the surface of *M. genitalium* for evading the neutralizing and interdicting effects of established antibody responses [35]; (2) intracellular invasion to escape attacks from cellular and humoral immunity and antibiotics [70]; (3) expression of immunoglobulin (Ig)-degrading enzymes for attenuating the humoral immune response [71]; (4) upregulation of detoxifying enzymes that degrade reactive oxygen species (ROS) and significantly increase the ability to counteract oxidative damage [72]; (5) formation of biofilms, which plays a role in the persistence of infection [73,74]; (6) secretion of nuclease for protecting against NET degradation [16,75]; and (7) inhibition of complements activation that contributes to the evasion of the host immune response [76]. The aforementioned mechanisms contribute to the survival and persistent colonization of the urogenital mucosae, which not only causes epithelial damage following chronic inflammatory stimulation, but also likely increases the possibility of transmission, the risk of transfer to the upper urinary tract, and susceptibility to other sexually transmitted infections [36,61] (Table 2).

**Table 2.** Putative factors for immune evasion and sustained survival of *M. genitalium.*

Virulence factors	Subcellular location	Function	Reference
P140	Transmembrane	Adhesion; Motility; Antigenic variation; Internalization	[19,24,28,70]
P110	Transmembrane	Adhesion; Motility; Antigenic variation; Internalization	[19,28,70]
MG428	Unclear	Recombination regulator	[77,78],
RecA (*mg339*)	Cytoplasm	Recombination regulator	[78,79],
RuvA (*mg358*)	Unclear	Recombination regulator	[78,80],
RuvB (*mg359*)	Unclear	Recombination regulator	[78,80]
RecU (*mg352*)	Cytoplasm	Recombination regulator	[81]
RrlA (*mg220*)	Transmembrane	σ accessory protein	[78]
RrlB	Unclear	σ accessory protein	[78]
MG281	Transmembrane	Protein M, Antibody-binding	[71,82]
Duf31 domain protein	Unclear	Cleavage of Igs (Predicted)	[82]
MG186	Cell membrane; Lipid-anchor	Nuclease, Degradation of NETs (Predicted)	[16,57]
MG408	Cytoplasm	MsrA; Defenses against oxidative stress	[45,83–85]
MG448	Cytoplasm	MsrB	[45,83,85]
MG454	Unclear	Ohr; Detoxification of oxidants	[72]
MG149	Cell membrane; Lipid-anchor	OsmC, Maintaining cellular integrity; Degrading ROS	[56]
MG427	Cytoplasm	OsmC, Detoxification	[72,86]

### Antigenic variation

Antigenic variation, also referred to as phenotypic switching, can be traced to genetic mutations. It is generally considered to occur more frequently than spontaneous mutations and has been confirmed in various human and animal mycoplasma species, including *M. genitalium*. It is considered to cause alterations in the antigenic properties of the surface components of pathogens that influence virulence potential. Antigenic variation is regarded as an effective mechanism for evading host immune surveillance via a potential mechanism for avoiding antibody clearance and is of great significance in *M. genitalium* survival [62,64,87]. In addition, antigenic variation can serve as an effective method to optimize adhesion for adaptation to changing hostile environments as well as transmission to new hosts [88,89]. Mycoplasmas exhibit antigenic variation by various methods, including on/off switching, size variation, domain shuffling, DNA recombination, gene conversion, and other types of variation involving gene or locus duplication. These genetic mechanisms can act independently or in tandem [89]. The relatively smaller genome of *M. genitalium* has limited space and does not encode proteins required for advanced maintenance mechanisms and repair systems for preventing deleterious mutations in essential genes. Therefore, *M. genitalium* has developed a sophisticated system for the antigenic and phase variation of major surface components, which is mediated by the combination of specific gene subsets via rapid and reversible genetic alterations for the generation of large cell-surface variants with a high likelihood of survival. The strategy utilized most frequently by *M. genitalium* for generating antigenic diversity is recombination [89]. Two types of gene variations have been established: (1) antigenic variation, related to the modification of surface structures, resulting in the formation of P110 and P140 variants in amino acid sequences [90] and (2) phase variation, a mechanism by which *M. genitalium* can regulate the expression of its immunodominant proteins [78,91]. As a result, spontaneous variation occurs in *M. genitalium* via a pathway in which P110 and P140 are not expressed, and this manifests as a reduction in its ability to attach to cultured cells or bind red blood cells (HA) [78,91].

#### Antigenic and phase variation of the major adhesins

P140 and P110 are the most immunological surface antigens surrounding the terminal organelle; both are capable of eliciting robust antibody responses in infected hosts. As a result, antigenic variation in these major adhesins is a crucial determining factor in *M. genitalium* host defence evasion and persistent infection [19,35]. At present, information on the antigen variation in *M. genitalium* is primarily based on these two immunodominant surface proteins encoded by the *mgpB* and *mgpC* genes in the MgPa operon [26]. The MgPa operon is composed of three transcriptionally linked genes (*mgpA, mgpB*, and *mgpC*) [35]. The expression site of the MgPa operon is present in only one copy but there are nine repetitive elements in the form of truncated copies of the *mgpB* and *mgpC* genes dispersed throughout the genome, which are referred to as MgPar sequences or MgPa repeats [26,41,90]. Although repetitive elements do not seem to generate protein-coding sequences directly, antigenic variation may occur via the recombination of these DNA repeats with the MgPa operon, resulting in antigenically distinct MgPa variants [19,92]. Interestingly, This reciprocal recombination is more commonly the cause of the corresponding full-length P140 and P110 amino acid sequence diversity, instead of premature truncation as a consequence of the insertion of stop codons [35]. As a haploid organism, *M. genitalium* can express only a single P140 and P110 isoform at a given time; thus, alteration of the gene sequences at their expression sites results in a distinct P140 and P110 on its surface. Due to their involvement in adhesion and cell division, the altered amino sequence in response to *in vivo* environmental changes and/or the availability of certain human cell types aids efficient cell adhesion, movement, cell division, and immune evasion [90].

In addition to protein heterogeneity, there is a wide range of intrastrain *mgpB* and *mgpC* diversity due to segmental recombination with MgPar donor sequences [8,35]. Furthermore, despite the fact that the MgPar regions encode homologs of *mgpB* and *mgpC*, the copies are non-identical and incomplete, these may serve as a reservoir for alternative *mgpB* and *mgpC* sequences [35]. This recombination can also occur between MgPars or between two distinct variable regions within the expression sites of *mgpB* and *mgpC* [93]. Although the recombination machinery is still lacking, few examples have indicated that non-reciprocal recombination or gene conversion are involved in recombination [93]. However, more and more evidence has shown a bias towards reciprocal recombination, as opposed to gene conversion in patient samples, *in vitro* growth of bacteria, as well as experimental animal models [35,91–94].

In addition to being a source of antigenic variation, the MgPars are implicated in a general phase variation mechanism, which can be switched on and off reversibly or irreversibly, resulting in variation in the adhesion capabilities of *M. genitalium*. Most phase variants arise via recombination-dependent mechanisms [19]. P140 and P110 phase variants have been characterized by Burgos et al, who showed that the lack of P140 and/or P110 expression was the result of large genomic deletions caused by a single cross-over between the *mgpB* or *mgpC* expression site and an adjacent MgPar that results in the elimination of intervening sequences [19,35]. Burgos et al. classified the phase variants into seven classes based on the nature of the mutation [91]. Most phase variants that do not express *mgpB* and/or *mgpC* are considered to be formed by recombination with MgPa repeat regions containing *mgpB* and *mgpC* variable sequences, and these variants are frequently reversible. For example, recombination between the expression site and MgPar sites may be related to two variable regions and the intervening conserved sequences. In this manner, the translocation of conserved *mgpB* and *mgpC* sequences to the participating MgPar site led to the formation of an incomplete *mgpBC* operon [91]. Other mechanisms of variance are considered to involve nonsense or frameshift mutations. It is widely accepted that most phase variants are reversible; however, irreversible phase variants also exist, including those formed by recombination between regions KLM of *mgpC* and MgPar5 with the subsequent deletion of the intervening sequences (class I), and by recombination between variable regions of *mgpB* and MgPar5, resulting in the deletion of portions of *mgpB* and *mgpC* (class II). Nevertheless, irreversible phase variants may still be reversed by the acquisition of exogenous DNA from other *M. genitalium in vivo* since the gene transfer event has been confirmed in certain mycoplasma species [91]. Whether the same is also present in *M. genitalium* needs further verification. Furthermore, the distribution and architecture of the repetitive sequences within the MgPar sites leading to *mgpB/C* phase variation may also be a mechanism for deletions in *mgpB/C* genes [79].

#### Variation of trinucleotide tandem repeats (TTRs)

TTRs are a specific type of repeats present in humans. A comprehensive analysis of the *M. genitalium* genome showed the presence of short tandem repeats in 18 loci, most of which were TTRs. The presence of TTRs was confirmed in several regions of *mgpB*, *mgpC*, and MgPars, and TTRs are variable in the repeat copy number as well as the repeat unit sequences among or within strains [95–97]. Ma et al. classified TTR variations in the repeat unit into three categories: (1) slipped-strand mispairing of TTRs in the *mgpB*-C and *mgpB*-F regions; (2) homologous recombination of TTRs in the *mgpB* and *mgpC* repeat regions as well as in some MgPars; and (3) site-specific recombination and other unidentified mechanisms in some regions of the MgPa operon and MgPars. Additionally, recombination may occur independently or in conjunction with slipped strand mispairing [96]. Moreover, since the repeat copy number appears to be a multiple of three nucleotides, changes in the copy number would not necessarily abrogate the reading frame or disrupt protein translation. Interestingly, the repeat copy number of TTRs can result in heterogeneity in the size of the poly (Ser/Thr) chain in the MgPa operon, potentially increasing the antigenicity of P140 and P110. Moreover, the functional significance of the Ser/Thr-rich repetitive motifs in the MgPa operon is currently unknown. Ser/Thr-rich repetitive motifs may function as flexible spacer regions to improve protein interactions or serve as sites for protein modifications such as glycosylation and phosphorylation, as has been found in other organisms [98,99]. TTR variation is most likely a representative mechanism for maximizing MgPa operon variation, acting as a complement of genetic variation associated with segmental recombination between MgPa and MgPars, which can enhance antigenic variation, and thus be helpful to *M. genitalium* in adhesion, colonization, and the ability of the organisms to evade the host immune system [96].

#### Factors involved in antigenic variation

##### 1. σ20 and the recombination system

M.*genitalium* has a basic recombination system involving RecA, RuvA, RuvB, and RecU. σ^20^ is an alternative sigma factor that controls the activation of homologous recombination and plays a significant role in the formation of P140 and P110 variants. Specifically, σ^20^ was reported to regulate the transcription of *recA* (*mg339*), *ruvA* (*mg358*), *ruvB* (*mg359*), and several genes with unknown functions [78,80], all of which are considered necessary to facilitate strand exchange, branch migration, and resolution of the Holliday junction, a cross-shaped structure formed during genetic recombination. For example, RecA exhibits DNA strand exchange activity *in vitro* in an ATP-dependent manner, which facilitates chromosomal rearrangement and DNA repair and helps maintain the genomic stability of *M. genitalium*. Its recombination activities are also affected by Mg^2+^, pH, and the presence of single-stranded DNA binding protein [78,79,100]. Unlike other bacterial species, in which RecA plays a limited role in DNA repair, RecA in *M. genitalium* is important for *mgpB* and *mgpC*–MgPar recombination, which results in antigenic and phase variation [79]. RecA also contributes to sequence diversity at the *mgpB/C* locus, and RecA deficiency results in a declining trend of variation in *mgpB* and *mgpC*; this illustrates the importance of RecA in antigenic variation [79,80]. In contrast, RuvA and RuvB are involved in processing the recombination intermediates that form as a result of RecA activity. Although there remains a lack of reports on the enzymatic machinery necessary to facilitate *mgpB/C* diversity in *M. genitalium*, RuvA and RuvB have been shown to act as branch migration proteins. The deletion of RuvA and RuvB impairs the ability to generate *mgpB* and *mgpC* phase and sequence variants, which confirms their importance in the production of antigenic variants [79,80]. In addition, the Holliday junction RuvAB, as the only branch migration pathway in *M. genitalium*, may be involved in the regulation of the degree of *mgpB* or *mgpC* sequence variation [80]. A hypothetical protein encoded by *mg352*, assumed to be a Holliday junction resolvase, may be pivotal for recombination in *M. genitalium*, since the homologous RecU in *M. pneumoniae* increases the incidence of homologous DNA recombination events in certain strains [81].

In addition to RecA, RuvA, and RuvB, recombination regulatory loci A and loci B (*rrlA* and *rrlB*), which are linked to homologous recombination, identified recently, are necessary for the activation of σ^20^, and severe recombination defects arise with the loss of expression of these genes. Additionally, the overexpression of σ^20^ can promote the transcription of *rrlA* and *rrlB*, which encode proteins involved in the feed-forward loop that stabilize and prolong the activity of σ^20^ and possibly affect homologous DNA recombination in *M. genitalium* [78].

##### 2.MG428

MG428 is a novel virulence factor of *M. genitalium* that acts as a positive regulator of recombination by coordinating the expression of critical recombination factors, including RecA, RuvA, RuvB, and ORF2. Although the function of OFR2 remains unknown, co-transcription with the Holliday junction protein RuvAB suggests that it may be involved in recombination [77,101]. The coordinated induction of these genes is closely related to an increase in *mgpB* and *mgpC* variation, indicating that MG428 activity is necessary to initiate *mgpB* and *mgpC* variation. MG428-deficient cells are unable to generate *mgpB* and *mgpC* variants despite normal RecA expression [77], which implies that RecA expression may be dependent on the coordination of other MG428-regulated factors. Intriguingly, the silencing of two MG428-regulated genes (*mg414* and *mg010*) exerted limited effect on *M. genitalium* recombination [101]; this implies that MG428 regulates various biological functions, in addition to recombination.

### Invasion into host cells and intracellular survival

Mycoplasma was considered to be an extracellular parasitic bacterium, prior to the discovery of its facultative intracellular nature. With the development of techniques such as confocal laser scanning microscopy, it is now possible to distinguish between invasion and adherence of the majority of pathogens. Intracellular parasitism and perinuclear targeting are considered crucial virulence characteristics that enable *M. genitalium* to establish persistent infection [70,102,103]. Perhaps the most striking example is its intracellular localization in vaginal and cervical epithelial cells, which may serve as a survival niche for *M. genitalium* and shield it from immune clearance, ultimately promoting its survival and the maintenance of chronic infection [59]. Although it has been established that the invasive process requires multiple factors, such as receptors, functional genes, kinases and cytoskeletal rearrangements, the kinetics of *M. genitalium* internalization and the details of its subcellular location remain unknown [70]. In the early stage of the *M. genitalium*–epithelial cell invasive process, certain alterations in host cell membrane function, loss of cell viability, and vulnerability of the nuclear membrane are known to occur, which lead to the intranuclear penetration of *M. genitalium*. Cell invasion appears to be mediated by the specialized terminal structure and specific enzymes, as the absence of P140 and P110 significantly reduces internalization and perinuclear/intranuclear localization [8,70]. In addition, the chaperone GroEL has been implicated in the invasion of *M. penetrans*. GroEL is produced by almost all mycoplasmas and likely to act as an adhesin-invasin, which indicates that it may be of significance in the invasion of *M. genitalium* [104]. The invasive ability of mycoplasmas in cell penetration varies and is dependent on cell types [8]. Certain conditions, such as persistent infection and appropriate temperature, significantly enhance the invasiveness of *M. genitalium* and increase its internalization into host cells. Studies have also suggested that invasion by mycoplasma is site-directed and receptor-mediated, similar to the infection process of *Chlamydia* [8]. Host DNA damage may occur in cells with persistent *M. pneumoniae* infection. As expected, *M. genitalium* invasion can cause DNA degradation and apoptosis-like morphological alterations, which are induced in part by the nuclease MG186. Thus, cell invasion by *M. genitalium* is a critical virulence factor for cytotoxicity, and helps establish an intracellular anchor for *M. genitalium* that protects it from the host immune response and antibiotic-mediated killing, thereby facilitating persistent infection [16].

### Cleavage of Igs

Igs are crucial for both innate and acquired immunity to protect the host from pathogen invasion. Many pathogenic bacteria have evolved the ability to subvert Ig-mediated killing and are consequently associated with sustained survival in the host. One such mechanism is the binding of the Ig Fc region by certain characteristic proteins of pathogenic bacteria, leading to the impairment of immune effector molecules. Another mechanism involves proteases that cleave Ig molecules at their hinge regions, thus decoupling the antigen recognition capability of the Fab region from clearance mechanisms elicited by the Fc region [105]. Many mycoplasmas, such as *Ureaplasma urealyticum and Ureaplasma parvum*, have been shown to contain a two-protein system known as the MIB-MIP system. The MIB-MIP system is composed of an Ig-binding protein (MIB) and an Ig protease (MIP), which are responsible for the capture and cleavage of IgG, respectively, and act as effective factors for evading the host immune system [82,106]. This indicates that *M. genitalium* may also possess similar strategies to evade or disrupt the immune response mediated by Igs. However, *M. genitalium* lacks the MIB-MIP system, but alternatively relies on protein M and Duf31, which appear to perform a similar function to the MIB-MIP system. Protein M is an MIB found in mycoplasmas such as *M. pneumoniae* and *M. gallisepticum*. Rajesh et al. showed that MG281, a functionally uncharacterized membrane-bound protein of 556 amino acids, is the protein M counterpart in *M. genitalium* [71,82]. The crystal structure of MG281 is unique among antibody-binding proteins in that it binds to both the λ and κ chains of antibodies with high affinity. One possible mechanism by which MG281 inhibits antibody-antigen binding is that it disrupts the complementarity-determining regions and/or uses the C-terminal domain to sterically obstruct the access to the antibody-binding site. In this regard, MG281 of *M. genitalium* may be important for host-bacteria combat and immune evasion [71].

Duf31, also an MIP-like protein, is widely present in pathogenic mycoplasmas, and important in evading host Ig-mediated defence. Duf31 was initially regarded as an MIP and MIP paralog, but has now been confirmed to be non-homologous to MIP. In *M. genitalium*, *duf31* annotated genes are found in multiple copies, and although the enzymatic function of *duf31* is unclear, its amino acid linear sequence suggests that it may exhibit the same serine protease activity as MIP [82]. Therefore, protein M and Duf31 cooperate to generate a novel pathway that shields mycoplasma from host Ig-mediated damage, facilitating its long-term survival for a longer time period in the host environment.

### Defences against oxidative stress

Mycoplasmas are inevitably exposed to oxidative stress during infection as a result of glycerol metabolism and host immune response. It has been demonstrated that various mycoplasmas can use glycerol as a carbon source during metabolism, producing H_2_O_2_ as a byproduct [107,108]. These metabolic processes are extremely deleterious to mycoplasma survival. Additionally, the activation of polymorphonuclear cells and monocytes/macrophages also results in the production of ROS and nitric oxide following mycoplasma infection, which exert a cytopathological effect on mycoplasma colonization [17,59]. While the production of these toxic products in *M. genitalium* requires additional validation, it is established that *M. genitalium* must possess detoxifying enzymes to counteract its toxic effects. Although typical detoxification enzymes or antioxidant stress proteins, such as SOD, Kat, and AhpC, have not been detected in *M. genitalium*, possibly because of its small genome size [72], the pathogen does produce some detoxifying enzymes, including methionine sulphoxide reductase (Msr) and organic hydroperoxide reductase (Ohr), which support its persistence in the host.

#### Msr

ROS have a proclivity for oxidizing methionine (Met) residues to Met sulphoxide (Met-O), which can cause the inactivation of functional proteins. Met-O comprises two stereoisomers, Met-S-O and Met-*R*-O. *M. genitalium* encodes two Msrs, MsrA (MG408) and MsrB (MG448), which have been shown to reduce Met-S-O and Met-*R*-O, respectively [45,83]. The reduction of Met-O to Met is considered essential for the detoxification of reactive oxygen intermediates [45]. MsrA primarily remains localized to the cytosol, with a molecular mass of approximately 25 kDa. Its shares 79% amino acid sequence identity with the protein of *M. pneumoniae*, and its N-terminal region may contain a conserved cysteine sequence that has been shown to be critical for enzymatic activity [84,109,110]. The disruption of MsrA in *M. genitalium* has been shown to decrease its virulence potential. For example, *M. genitalium* lacking MsrA was less capable of adhering to sheep erythrocytes and incapable of surviving in hamsters; a plausible reason for this is that the external Met residues of the major cytadhesin P110 can be oxidized by oxidative agents. In this respect, MsrA is necessary for maintaining the function of adhesins and attachment organelle-related proteins in *M. genitalium* [84,85]. Additionally, the MsrA mutant strain was less cytotoxic to HeLa and C33A cells, more susceptible to THP-1 cell phagocytosis, and less capable of inducing THP-1 cell aggregation and differentiation than the wild-type strain [45]. This is the most appropriate illustration of the greater susceptibility of the MsrA mutant to phagocytosis and oxidative killing by phagocytes.

MsrB has been widely studied in *Neisseria gonorrhoeae* and *Escherichia coli*, but has a lower capacity for reducing free Met-O than MsrA [85,109]. Although MsrB in *M. genitalium* has also been annotated, with 85.8% and 69.4% homology with those in *M. pneumoniae* and *M. gallisepticum*, respectively, there is limited information on the actual effect it exerts.

#### Ohr

Ohr and osmotically inducible protein C (OsmC) belong to the OsmC superfamily and share similar structures and functions. Generally, OsmC and Ohr are both cytoplasmic and membrane proteins. Ohr is a thiol peroxidase containing two highly conserved cysteine residues linked by peroxide reduction, indicating that the primary function of Ohr is to protect organisms from the toxicity of organic H_2_O_2_. In some cases, Ohr plays the same role as OsmC proteins in the detoxification of organic peroxides [72,86,111]. It was recently demonstrated that putative MG454 is involved in the detoxification of oxidants generated by *M. genitalium* during metabolism. Apparently, the MG454 mutant strain was found to be sensitive to *t*-butyl hydroperoxide and cumene hydroperoxide, indicating that MG454 is biased towards Ohr rather than OsmC; MG454 expression is unresponsive to oxidative stress, but the mRNA is upregulated in response to physical stress, such as salt (NaCl) and heat, implying that *M. genitalium* lacks oxidative stress response regulation factors to modulate MG454 expression. Nonetheless, since the operation of σ factors as regulators under physical stress has been demonstrated in *Bacillus subtilis* [72], we cannot rule out the possibility that MG454 expression is controlled by a σ factor.

#### Osmotically inducible protein

MG149 is an osmotically inducible lipoprotein of *M. genitalium* that plays an important role in maintaining cellular integrity. The MG149 content was shown to increase significantly under physiological hyperosmolarity and change reversibly upon the relieving of osmotic stress. DNA supercoiling has been implicated in MG149 expression control [56]. It is of additional interest that MG149 is the predominant active component of *M. genitalium* lipoproteins that activates TLRs expressed on leukocytes, thereby contributing to the inflammatory response [56,112]. Although there is evidence that MG149 is capable of degrading ROS, the precise role of MG149 in protection against oxidative stress needs further investigation.

MG427 is another osmotically inducible protein required for *M. genitalium* growth *in vitro*. MG427 is primarily present in the cytoplasmic fraction, and only a minor part is present in the membrane fraction. MG427 was shown to be redox-active and decrease the toxicity of peroxides. The increased sensitivity to H_2_O_2_ and hypersensitivity to tert-butyl hydroperoxide in MG427 mutant strains suggested that MG427 is critical for defence against oxidative stress damage. Alternatively, the growth curves of MG427 mutant and wild-type *M. genitalium* strains in medium supplemented with either glucose or glycerol revealed that the MG427 mutant strain shows slow growth, implying that MG427 provides protection against oxidative stress-induced damage when H_2_O_2_ is produced as a byproduct of glycerol utilization. Furthermore, experiments demonstrated that recombinant MG427 reacts with dithiothreitol and is capable of reducing organic hydroperoxides and H_2_O_2_ in a concentration-dependent manner, indicating that MG427 acts as a hydroperoxide peroxidase. Unlike MG454 expression, MG427 expression is inhibited by osmotic shock, ethanol, and heat shock, although the regulatory role of heat shock is relatively weak [86].

### Formation of biofilms

Both bacteria and fungi form biofilms that facilitate their survival in hostile environments and provide protection against biotic and abiotic stress [73]. Bacterial biofilms are composed of bacteria encased in an extracellular matrix consisted of polysaccharides, polypeptides, nucleic acids, and lipids that provide protection to the bacteria. The matrix of the biofilm is composed of polymeric substances secreted by bacteria within the biofilm. Extracellular polymeric substances (EPS), which are important for the composition of the matrix, are mainly composed of poly-*N*-acetylglucosamine in some bacteria capable of forming biofilms. *M. genitalium* strains contain a high concentration of N-acetylglucosamine, indicating the presence of biofilm-specific EPS [74,113]. Three steps are involved in the formation of a biofilm *in vivo*: (1) attachment to accessible host proteins owing to the hydrophobic nature of microbial surfaces or particular bacterial surface molecules; (2) anchored growth and colonization; and (3) detachment and dissemination by sheer mechanical forces (blood flow), degradation of extracellular matrix, and nutrient limitation for the formation of mature biofilms [73]. Biofilms are considerably more resistant to stress, including heat and desiccation, than planktonic cells, and play crucial roles in the resistance to antibiotics, antibodies and phagocytes, allowing the pathogen to circumvent host defences and cause persistent infections. Another detriment of biofilms is that they release planktonic cells periodically, leading to bouts of acute infection [73,113]. Scanning electron microscopy shows that the bacterial tower matures gradually during biofilm formation, and mature *M. genitalium* biofilms tend to exhibit the disappearance of polarization. Growth index analysis revealed that the viability and metabolism decrease in cultures with disrupted biofilms in response to antibiotic treatment compared with that in cultures with intact biofilms, indicating that biofilms can be attributed to the survival of *M. genitalium* and contribute to the perseverance of this bacterium [74].

### Degradation of NETs

Neutrophils are the first line of defence against pathogen infection in the innate immune system. Apart from neutrophil phagocytosis and antimicrobial secretion, the release of NETs has been considered as a novel strategy for bacterial elimination [114]. NETs are web-like structures comprised of DNA, nuclear proteins, and granular enzymes released during non-apoptotic cell death after neutrophil activation and cell membrane disruption. The formation of NETs is a highly effective strategy for entrapping and killing invading pathogens, such as bacteria, fungi, and parasites [57,114,115]. Interestingly, various microorganisms are capable of inhibiting, degrading, or resisting the destructive effects of NETs, and the mechanisms used are as follows: (1) suppressing host inflammatory responses to inhibit NET release, (2) secreting nucleases to degrade the DNA components of NETs, and (3) developing resistance mechanisms against the microbicidal components of NETs [116]. Neutrophils appear to be less important for mycoplasma infection than macrophages, as the co-incubation of neutrophils and mycoplasmas exerts no inhibitory effect on mycoplasma growth. However, certain mycoplasmas utilize various nucleases to degrade the DNA components of NETs. MnuA was found to be a non-specific nuclease in *M. bovis* and was shown to degrade NETs rapidly *in vitro*. Homologous genes were also found in *M. pneumoniae* and *M. genitalium* [117]. In *M. pneumoniae*, Mpn491 is a secretory nuclease that has been shown to degrade NETs, and the absence of Mpn491 enhances the susceptibility to neutrophil killing [57]. In parallel, MG186 is a nuclease produced by *M. genitalium* that exhibits sugar-non-specific endonuclease and exonuclease activity, helps degrade host nucleic acids, and serves as a source of nucleotide precursors for survival [16]. Therefore, it is reasonable to believe that MG186 may perform similar functions in the degradation of NETs. However, the precise nature of its function remains unknown.

### Inhibition of complement activation

Complement factors are important components of innate immunity that are essential for host defence against infections, elimination of cellular debris, and response to inflammatory processes. Factor H is a negative regulator of the complement system, which can interact with receptors on host cells and has been implicated in the restraint of unanticipated complement activation. Certain mycoplasmas have evolved strategies to disrupt the activity of factor H, and can thus inhibit complement activation and enhance their chances of survival [76]. For example, EF-Tu, P146, and P46 of *M. hyopneumoniae* are factor H-binding proteins. Through this interaction, C3 deposition on the *M. hyopneumoniae* surface is reduced to prevent subsequent complement activation. Hence, the binding of factor H aids the evasion of *M. hyopneumoniae* from complement killing and promotes adherence to epithelial cells. Indeed, *M. genitalium* has been shown to hijack factor H and present itself as host tissue, which is important for avoiding complement attack and, as a result, contributes to the evasion of the host immune response.

## CANDIDATE VIRULENCE FACTORS

### Glycosyltransferase

M.*genitalium* requires precise regulation of membrane fluidity and integrity because it lacks a cell wall. Glycoglycerolipids are critical structural components of their unique plasma membranes, contributing to their bilayer properties and stability. However, because certain glycoglycerolipids are absent in parasitic host cells, their biosynthesis by glycolipid synthase is considered crucial for mycoplasma pathogenesis in infection. Indeed, *M. genitalium* encodes a unique glycosyltransferase, MG517, which catalyzes the transfer of glucosyl or galactosyl residues to membrane-bound diacylglycerol, facilitating the formation of membrane glycoglycerolipids [118–120]. MG517 has an estimated 70% homology with *M. pneumoniae* MPN483. As a membrane-associated protein [118], a short α-helix was detected at the top C-terminal of MG517 with clear amphipathic character, which plays an important role in guiding MG517 to anchor to the cell membrane. Truncation of a part of the helix significantly reduced the synthesis of glycoglycerolipids, suggesting that the helix structure is essential for enzymatic activity. As expected, the inhibition of MG517 delayed the growth of *M. genitalium* [118,119]. In *M. pneumoniae*, glycolipids are critical pathogen-associated molecular patterns for pathogen recognition by the immune system [121]; however, it remains unknown whether *M. genitalium* exhibits similar pathogenic activity.

### Serine/threonine kinase (STK) and serine/threonine phosphatase (STP)

To adapt to its changing environment, *M. genitalium* has evolved a series of signal transduction systems that regulate gene expression via protein-protein interactions. In some pathogenic bacteria, such as *Yersinia*, *Mycobacteria*, and *Pneumococcus*, STK and STP are considered as virulence factors. It is well established that the loss of STK or STP appears to decrease the viability and increase the load of some bacteria. *M. genitalium* also expresses STK, which is encoded by *mg109*, and STPs, which are encoded by *mg108*, *mg207*, and *mg246*. MG207 is an alkaline phosphatase that is Mg^2+^-dependent and capable of dephosphorylating threonine phosphate. The MG207 mutant strain was found to be less cytotoxic to HeLa cells owing to lower H_2_O_2_ production caused by the suppression of its glycerol utilization capacity and the inability to differentiate THP-1 cells; these factors led to an overall reduction in the virulence of *M. genitalium* [122,123]. In *M. pneumoniae*, mutation of serine/threonine protein kinase C (PrkC) results in non-adherent growth and loss of cytotoxicity. One possible explanation is that the phosphorylation of PrkC-regulated proteins (HMW1, HMW2, and HMW3) is essential for the stability of cytadherence [124]. The lack of MG207 expression in *M. genitalium* also led to the differential phosphorylation of proteins, including MG407 and MG317, which play a role in ATP synthase and carbon source metabolism as well as in gene expression and cytadherence [125]. The role played by MG207 in the pathogenesis of *M. genitalium* remains unclear, and further research is warranted to confirm this.

### Hydrogen sulphide

A putative cysteine desulfurase encoding gene, *mpn487*, which has been designated as *hapE*, was recently identified in *M. pneumoniae*, and the protein was shown to be capable of generating alanine, pyruvate, and hydrogen sulphide [126]. Hydrogen sulphide can cause haem modification and erythrocyte lysis, which provides additional insights into the virulence mechanisms of *M. pneumoniae*. Iron acquisition is a critical growth-limiting factor for most pathogens in the host environment, and haemolysis is one of the strategies used to obtain iron from the host. *M. pneumoniae*, conversely, can thrive in the absence of iron, suggesting that in *M. pneumoniae*, haemolysis serves as a different approach for the acquisition of nutrients, such as amino acids and nucleotides, rather than iron [126]. Of note, because the *M. genitalium mg336* gene shows high homology with the *hapE* gene of *M. pneumoniae*, we cannot rule out the possibility that MG336 plays a similar role to HapE. However, further research is required to fully understand whether MG336 acts as a potential virulence factor for *M. genitalium*.

### Lipolytic enzymes

Lipolytic enzymes, such as lipases, esterases, and phospholipases, are expressed by some mycoplasmas, such as *M. gallisepticum* and *Acholeplasma laidlawii*, which are necessary for mycoplasma metabolism and pathogenicity. Because traditional fatty acid-degrading enzymes are absent in these pathogens, lipolytic enzymes may play a key role in the nutritional requirements for long-chain fatty acids [127]. *M. hyopneumoniae* P65 is a lipolytic enzyme capable of degrading short-chain fatty acids originating from the host cell membrane to generate energy. Exogenous fatty acids are generated by lipases related to the synthesis of lipoproteins, phospholipids, and glycolipids, and thus play a role in mycoplasma metabolism. The process of nutrient acquisition is associated with mycoplasma pathogenicity [127]. Certain lipase orthologous genes were shown to be present in the complete genomic sequences of *M. genitalium*, but their precise role in pathogenesis is yet to be understood [1].

### Dihydrolipoamide dehydrogenase

Dihydrolipoamide dehydrogenase is an E3 member of the PDH complex. Dihydrolipoamide dehydrogenase-mutant *M. gallisepticum* showed decreased virulence. Owing to the lack of experiments correlating the function of the dihydrolipoamide dehydrogenase gene in *M. genitalium* [128], we hypothesized that the absence of dihydrolipoamide dehydrogenase reduces virulence, similar to that observed in *M. gallisepticum*.

## CO-INFECTION

M.*genitalium* frequently co-infects with other pathogens, such as HIV, *C. trachomatis*, and *N. gonorrhoeae* [129,130]. It is of additional interest that infection by *C. trachomatis, N. gonorrhoeae*, or *T. vaginalis* increases the chance of infection by *M. genitalium* [58,131]. Although the precise mechanism by which *M. genitalium* causes co-infection is unknown, persistent colonization of the urogenital mucosae may play a significant role in co-infection. These risk factors, which contribute to long-term inflammation, may cause epithelial damage and potentially enhance the susceptibility to other sexually transmitted infections [61]. Considering *M. genitalium* and HIV co-infection as an example, to begin with, acute *M. genitalium* infection induces an immune response that involves the secretion of cytokines, chemokines, and other antimicrobial defence factors, thereby increasing the susceptibility to HIV infection. Additionally, IL-7 secretion induced by *M. genitalium* infection was shown to increase the likelihood of HIV entry into thymocytes and promote HIV replication *in vitro* [58]. Furthermore, the recruitment of targeted cells, such as CD4+ T lymphocytes and macrophages, to the lower reproductive tract mucosa following *M. genitalium* infection allows HIV to pass through the mucosal barrier, reach the subepithelium by adhering to these vulnerable cells and replicate, which may further increase the susceptibility to HIV infection [58,132].

## MALIGNANT POTENTIAL

In the 1960s, mycoplasma-infected cell cultures sparked speculation about the link between mycoplasma and cancer. This speculation was based on the presence of antibodies against mycoplasma in leukaemia patients and infection of cell cultures with mycoplasma [133,134]. Although certain strains of mycoplasmas, such as *M. hyorhinis, M. hominis, M. penetrans*, and *M. salivarium*, can be detected in cancer patients [135,136], reports focusing on *M. genitalium*-detection in cancer patients are rare. Even for the same patients, antibody and DNA tests for *M. genitalium* frequently yield inconsistent results [137,138]. Although Biernat-Sudolska et al. confirmed that certain mycoplasma infections may increase the risk of human papillomavirus infection, *M. genitalium* does not do so [139]. In light of the prevalence of *M. genitalium* infections as well as the evolution of resistance strains, the epidemiological evidence for a definitive explanation of *M. genitalium* infection and tumourigenesis has remained contentious [18]. During the lengthy process of chronic infection and persistent inflammatory stimulation, it is impossible to overlook this seemingly inconsequential effect. Even mycoplasmas with low virulence can influence the phenotype of mammalian host cells in the presence of persistent infection, implying that chronic stimulation by some mycoplasmas may enhance the host cell sensitivity to transformation events and accelerate tumour development in mammalian cells [140,141]. In 2009, Namiki et al. reported for the first time that *M. genitalium* infection can cause the malignant growth of a benign prostate cell line [140]. The authors showed that after 19 weeks of infection, cells attained anchorage-independent growth and also showed improved migratory and invasive abilities. Both the increased migration and invasiveness of infected cells as well as the xenograft model in immune-compromised mice can be described by nuclear atypia and active mitosis, indicating that the infected prostate cells may have undergone malignant transformation. Interestingly, tumours derived from *M. genitalium*-infected BPH-1 cells expressed high levels of cell-surface tumour markers, such as Ki-67, a proliferative marker indicative of malignant transformation. These changes were associated with the accumulation of chromosomal aberrations and polysomy (the addition of extra complete chromosomes), suggesting that, for the first time, *M. genitalium* infection was shown to exhibit malignant potential in benign epithelial cells [140]. As another novel finding, Zella et al. reported that *M. fermentans* DnaK can disrupt several cellular pathways, including those involving DNA-PK and PARP1, both of which are essential for effective DNA repair, as well as USP10, a p53 regulator involved in p53-dependent anticancer activities [131]. Although it is unknown whether DnaK exerts a similar effect in *M. genitalium*, the homology between *M. genitalium* and *M. fermentans* is up to 55.7%. The precise mechanism underlying carcinogenesis by *M. genitalium* DnaK is yet to be determined, and further investigation is necessary.

## FUTURE PERSPECTIVES

M.*genitalium* has adopted a parasitic mode of existence and is confronted with complicated host environments that it must adapt to for survival. The development of molecular biology and imaging technology has made it easier to understand the virulence in mycoplasma-host interactions and the pathogenesis of *M. genitalium*. In the past 20 years, findings from different studies have advanced our understanding of *M. genitalium* cytadherence. However, certain glycolytic enzymes can also function as mycoplasma adhesins involved in the pathogenesis of this bacterium, implying that besides the tip-mediated cytadherence of *M. genitalium*, other adherence pathways may be required for efficient cytadherence. Despite the identification of a series of proteins related to motility, the exact mechanism of *M. genitalium* motility warrants investigation. The method by which the terminal organelle acts as a power provider and regulates the transmission of movement may also be an interesting topic for future investigations. Elucidating the mechanism underlying the gliding movement is critical, as it plays a crucial role in the spread of infection and enhances the ability of the pathogen to access target cells.

Because *M. genitalium* infection is usually asymptomatic; a lack of secretory exotoxins, such as CARDS toxin, could explain its apparently relatively low virulence compared to that of *M. pneumoniae*. The question is, under what conditions do the obvious clinical symptoms appear after *M. genitalium* infection? A plausible explanation is that it depends on the organism burden, as endocervical epithelial cells inoculated with a high dose of *M. genitalium* demonstrated distinct cell lysis, detachment, or growth inhibition, whereas no microscopic or quantitative differences were observed in cultures with lower microbial loads [61]. Another possibility is that there exist several different biovars of *M. genitalium*, each of which may exhibit varying degrees of pathogenicity through the secretion of unknown toxins, such as CARDS toxin, thereby inducing distinct clinical symptoms. Furthermore, the internal physiological environment may also affect the activity of *M. genitalium*. The activity of *M. genitalium* nuclease MG186 is known to be influenced by Ca^2+^, whereas the Ca^2+^ concentration is significantly affected by the physiological conditions of the host body, and under different environmental stimuli, the fluctuation of Ca^2+^ concentration can be transient, oscillatory, or sustained. Therefore, the internal environment of the host cell seems to affect the growth of *M. genitalium*. Importantly, several challenges remain in the identification of new virulence factors and differentially expressed genes in patients with different symptoms.

The mechanisms underlying persistent *M. genitalium* infection have been discussed extensively. It is conceivable that antigenic variation is an important contributor to the evasion of host immune responses. However, apart from the phenotypic plasticity of the surface antigens P140 and P110, it remains unclear whether other surface-exposed lipoproteins of *M. genitalium* can exhibit similar recombinational variation, or whether there are new types of antigenic variation that need further exploration. Notably, the significance of invasion and intracellular survival in pathogenesis remains unclear. Currently, it is unclear whether cell invasion after *M. genitalium* adhesion is mediated by receptors, and the signalling pathways in subsequent events merit further investigation.

Another topic of interest is the existence of triacylated lipoproteins. Because *M. genitalium* lacks an *Lnt*-encoding gene capable of encoding the enzyme responsible for *N*-acylation, the mechanism underlying the acylation of cysteine residues remains unknown. However, the discovery of the triacylated lipoprotein MG040 in *M. genitalium* suggests that an unknown functional protein performs the same function as the *Lnt* enzyme in *M. genitalium*; therefore, a complete understanding of this functional gene will be important in future as well. In conclusion, the progressive refinement of our knowledge of the functional genes of *M. genitalium* will not only help comprehend how the prokaryotic microorganism with the smallest genome utilizes its limited genes to exhibit pathogenicity and survival with maximum efficiency, but also provide important references for the development of drugs or vaccines.

## Data Availability

Data sharing is not applicable for this article, as no new data were created or analysed in this study.
